# Identification of novel biomarkers for prediction of neurological prognosis following cardiac arrest

**DOI:** 10.18632/oncotarget.14877

**Published:** 2017-01-28

**Authors:** Jung Woo Eun, Hee Doo Yang, Soo Hyun Kim, Sungyoup Hong, Kyu Nam Park, Suk Woo Nam, Sikyoung Jeong

**Affiliations:** ^1^ Department of Pathology, Functional RNomics Research Center, Cancer Evolution Research Center, College of Medicine, The Catholic University of Korea, Seoul, Republic of Korea; ^2^ Department of Emergency Medicine, College of Medicine, The Catholic University of Korea, Seoul, Republic of Korea; ^3^ Department of Emergency Medicine, College of Medicine, The Catholic University of Korea, Daejeon, Republic of Korea

**Keywords:** peripheral blood transcriptome, molecular signature, neurological prognosis, cardiac arrest, cerebral performance category, Pathology Section

## Abstract

**Background:**

Early prognostication of neurological outcome in comatose patients after cardiac arrest (CA) is important for devising patient treatment strategies. However, there is still a lack of sensitive and specific biomarkers for easy identification of these patients. We evaluated whether molecular signatures from blood of CA patients might help to improve the prediction of neurological outcome.

**Methods:**

We examined 22 comatose patients resuscitated after CA and obtained peripheral blood samples 48 hours after CA. To identify novel blood biomarkers, we aimed to measure neurological outcomes according to the Cerebral Performance Category (CPC) score at 6 months after CA and to determine blood transcriptome-based molecular signature of poor neurological outcome group.

**Results:**

According to the CPC score, 10 patients exhibited a CPC score of one and 12 patients, a CPC score four to five. Blood transcriptomics revealed differently expressed profiles between the good outcome group and poor outcome group. A total of 150 genes were down-regulated and 237 genes were up-regulated in the poor neurological outcome group compared with good outcome group. From the blood transcriptome-based signatures, we identified that *MAPK3, BCL2* and *AKT1* were more specific and sensitive diagnostic biomarkers in poor neurological outcome with an area under the curve of 0.867 (*p*<0.0001), 0.800 (*p*=0.003), and 0.767 (*p*=0.016) respectively.

**Conclusions:**

We identify three biomarkers as potential predictors of neurological outcome following CA. Further assessment of the prognostic value of transcriptomic analysis in larger cohorts of CA patients is needed.

## INTRODUCTION

Sudden cardiac arrest (CA) remains an important cause of morbidity and mortality, although the overall outcome has improved recently through better emergency care, including early cardiopulmonary resuscitation (CPR), early defibrillation, and implementation of post-resuscitation care bundles [1, 2]. And also, many patients who have restoration of spontaneous circulation (ROSC) remain comatose. In comatose survivals, neurological outcome prediction is important for treating clinicians when making appropriate treatment decisions and counseling families about the withdrawal of life-sustaining therapies [3]. To improve the accuracy of prognosis prediction in those patients, the combination of several prognostic tools with clinical examination are clinically recommended [4]. Neurological examination remains the first step, and other tools, including electroencephalogram (EEG), electrophysiological examinations, brain imaging, and serum biomarkers of brain damage like neuron-specific enolase (NSE) and S-100B are used [5, 6]. But these tools are required appropriate skills and experiences for accurate interpretation and, especially, imaging studies have not been fully standardized and are subject to inter observer variability [6–8]. Moreover, since targeted therapeutic hypothermia (TTH) was shown to effectively improve the neurological outcome of comatose cardiac arrest survivors, TTH has become the standard of care for these comatose patients. As TTH is combined with several drugs, like sedative drugs and muscle relaxants and hypothermia, it affects metabolism of these drugs and decreases reliability of a clinical examination of those patients. TTH makes the prognostication of neurological outcome more complex [6].

Of those several prognostic tools, biomarker testing requires less bedside expertise, may be less confounded by sedatives and is more readily repeated. With these advantages of biomarkers, studies of biomarkers like NSE, S-100B and neurofilament heavy chain levels have been performed about the usefulness as a predictor for neurological outcome. But, they showed that different serum levels of biomarkers correlate with poor outcomes in each study. Besides, standardization and optimal timing of each biomarker have yet to be determined [9–13]. Hence, there is increasing interest in biomarkers, which have more sufficient sensitivity and specificity to be clinically useful.

In general, prior to the onset of dysfunctional effects in brain injury, the effects are already present at the cellular level. Effects at the cellular level depend on the intensity and duration of ischemia-reperfusion in the brain, and could be reflected by the activations of different pathogenesis of brain injuries. At the onset of cardiac arrest, genome expression, molecular and cellular changes develop and biomarkers reflecting these pathological mechanisms will be recognized as potent predictors for neurological outcome from CA.

The aim of this study was to evaluate whether molecular signature of peripheral blood from comatose patients after CA may help to find as a useful biomarker to predict neurological outcome.

## RESULTS

### Characteristics of patients

**Figure 1:**
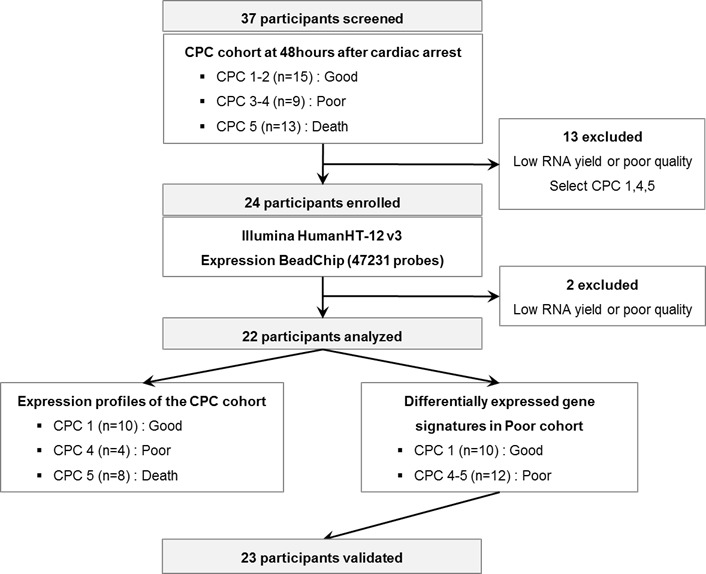
A schematic view of the procedure which contained patient cohorts and technologies used to find novel biomarkers in peripheral blood of CA patients The CPC cohorts at 48 hr after CA were separated to CPC 1-2, 3-4, and 5 and the blood transcriptome scans of CA patients were performed about selected patient sample. After the blood transcriptome scan, the data were analyzed to two different groups: Expression profiles of the CPC cohort and differentially expressed gene signatures in Poor cohort.

We investigated whether molecular signature of peripheral blood from early stage CA patients reflects different pathogenesis of CA, providing novel windows of gene expression changes as unique molecular signatures for CA. Early CA patients who remained comatose 48 hr after CA were selected for transcriptome analysis. Figure 1 provides an overview of this study design. The initial cohort consisted of 37 comatose patients who were successfully resuscitated from CA (CPC score 1-2 [*n* = 15], CPC score 3-4 [*n* = 9], CPC score 5 [*n* = 13]). Of these patients, 13 patients were excluded from the initial dataset due to lack of definite CA signatures. Two patients with CPC score 4 were excluded because of poor RNA quality or low RNA yield. Finally, 22 participants were dichotomized into good neurological outcome, CPC 1 (*n* = 10), and poor neurological outcome CPC 4-5 (*n* = 12), were analyzed on whole genome expression microarray to profile post-CA with its innate characteristic molecular signature.

Detailed demographics of enrolled patients are summarized in Table 1. There were no differences between the two outcome groups with regard to age, sex, initial cardiac rhythm, and preexisting diseases. Witnessed arrest, a shockable initial rhythm, the cardiac cause of CA etiology, the shorter time from arrest to ROSC, the lower score of APACHE II and the lower level of NSE at 48 hr after CA favored good outcome group. Significant differences in the presence of a witnessed arrest, the time from arrest to ROSC, APACH II and NSE were observed between the good and poor neurological outcome groups.

**Table 1:** Patient characteristics

	Good outcome	Poor outcome	*p*-value
	(n=10)	(n=12)	
Male, n (%)	8 (80)	11 (91.7)	0.42
Age, y, median (Q1-Q3)	57.00 (56.13-56.25)	55.50 (56.03∼56.25)	0.86
Witnessed, n (%)	10 (100)	10 (83.3)	0.00
Initial rhythm, n (%)			0.89
VF/VT	9 (90)	7 (58.3)	
PEA	1 (10)	2 (16.7)	
Asystole	0	3 (25.0)	
Primary cardiac cause, n (%)	10 (100)	9 (75.0)	0.22
MI	4 (40)	4 (33.3)	1.00
Time from arrest to ROSC, min,	20.00 (14.50∼40.75)	36.00 (33.20∼33.25)	0.03
median (Q1-Q3)			
Medical history, n(%)			
coronary disease	2 (20)	4 (33.3)	0.78
arrhythmia	1 (10)	1 (8.3)	0.89
hypertension	4 (40)	5 (41.7)	0.50
diabetes	0 (0)	3 (25.0)	0.22
lung disease	0 (0)	1 (8.3)	0.99
renal disease	0 (0)	1 (8.3)	0.99
APACHE II, mean (IQR)	15.50 (15.75-17.00)	33.00 (32.75-33.60)	<0.01
GCS 48 hr, mean (IQR)	3.00 (4.50-5.75)	3.00 (4.50-7.10)	0.648
NSE 48 hr, ng/ml, mean (IQR)	25.38 (22.16-23.66)	37.89 (46.37-63.20)	0.028

VF/VT, ventricular fibrillation/ventricular tachycardia; PEA, pulseless electrical activity; ROSC, return of spontaneous circulation; APACHE II, Acute Physiology and Chronic Health Evaluation II; GCS, Glasgow Coma Scale; NSE, neuron-specific enolase. Data are presented as mean with interquartile range, or number with percentages.

### Blood transcriptome scans differentiated cerebral performance category score at 48 hr after cardiac arrest

**Figure 2:**
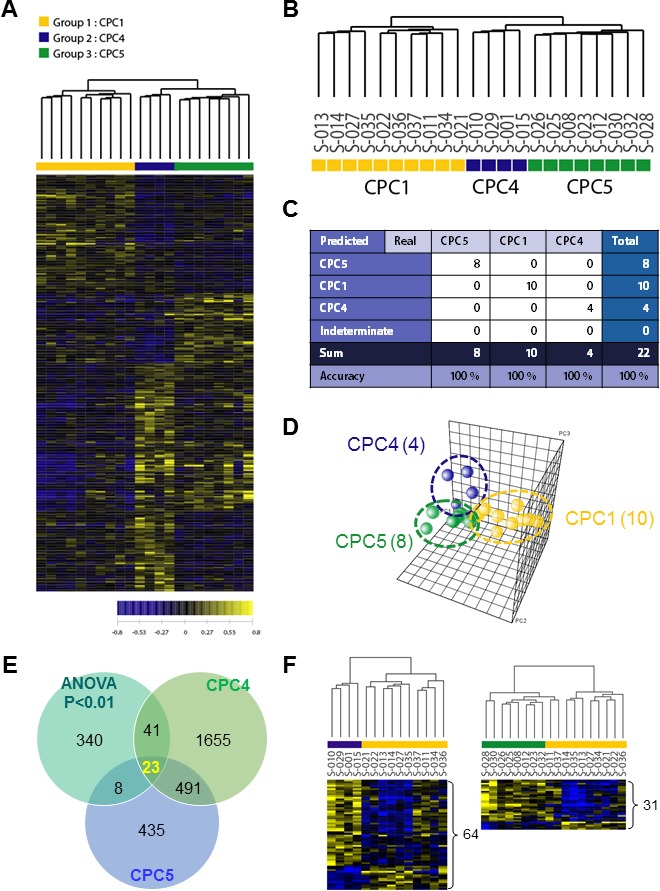
Hierarchical clustering analysis of expression profiling and determination of high-accuracy gene classifiers in CA patients **A**. Two-dimensional diagram of differential expressions of 412 genes and the data were organized by transcript and CPC category based on similarity. **B**. Dendrogram was derived from clustering using the 412 gene set. To identify classifier genes in the each CPC groups, the expression data of 22 specimens were subjected to the One Way ANOVA test (*p* < 0.01). **C**. Accuracy was tested by using the leave-one-out cross validation (LOOCV) method, and all tested-22 samples were categorized to three different CPC categories. **D**. PCA analysis of the 412 genes in each CA patient samples. The yellow sphere circle indicates the CPC 1 group, the blue circle indicates the CPC 4 group; the green circle indicates the CPC 5 group. **E**. Venn diagrammatic analysis of common gene signatures between CPC 4 and CPC 5 group. **F**. The dendrograms were obtained 64 genes of CPC 4 group and 34 genes of CPC 5 group. The heat-map showed supervised hierarchical clustering of recapitulated gene signature performed by using Genplex software; Pearson correlation, median centering, and complete linkage were used for all clustering applications.

Largely, transcriptomic regulation is driven by dynamic intracellular responses to a biological stimulus. Thus, to explore whether the large-scale gene expression change in peripheral blood reflects different pathogenesis of CA and to identify differentially expressed gene set of each CPC group, one way ANOVA test and hierarchical clustering analysis were performed. As shown in Figure 2A, hierarchical clustering analysis of the 22 samples with 412 outlier genes that passed ANOVA test (*p* < 0.01) resulted in two main clusters within dendrogram. One cluster (left; yellow) contained all of the CPC 1 group, and a second cluster (right; navy and green) contained all of the CPC 4 and the CPC 5 (Figure 2B). This result indicated that large-scale gene expression changes of early stage CA patients and such gene expression changes might serve as unique molecular signature for the diagnosis or prediction of CA. To retrieve 412 outlier genes that precisely discriminate each CPC, we used one way ANOVA-Bonferroni multi-classification algorithms, followed by whole computation (gene selection algorithm, the ratio of between-group to within-group sums of squares [BSS/WSS]). Then, these 412 outlier genes were further validated by prediction confidence analysis, the leave-one-out cross validation (LOOCV). A summary of the frequencies of class assignments using high-accuracy classifier (412 genes) is provided in Figure 2C with 100% of prediction for each 3 different class. As shown in Figure 2D, PCA of the 412 gene expressions was carried out for the correlation present in the multi-attributes. To compare differentially expressed genes between CPC 4 and CPC 5, we further analyzed recapitulated genes by Venn diagrammatic gene selection method. As depicted in Figure 2E, genes that were differentially expressed compared to CPC 1 were 64 and 31 in CPC 4 and CPC 5 group respectively, and displayed as heat-map (Figure 2F). Among these, 23 genes were identified as common genes in both CPC 4 and CPC 5 group.

### Identification of predictive molecular signatures and their biological processes based on the CPC transcriptomics

In addition, we sought to identify differentially expressed gene set of each poor CPC score group compared to good CPC score group by using Welch's *t* test as described in Materials and Methods.

**Figure 3:**
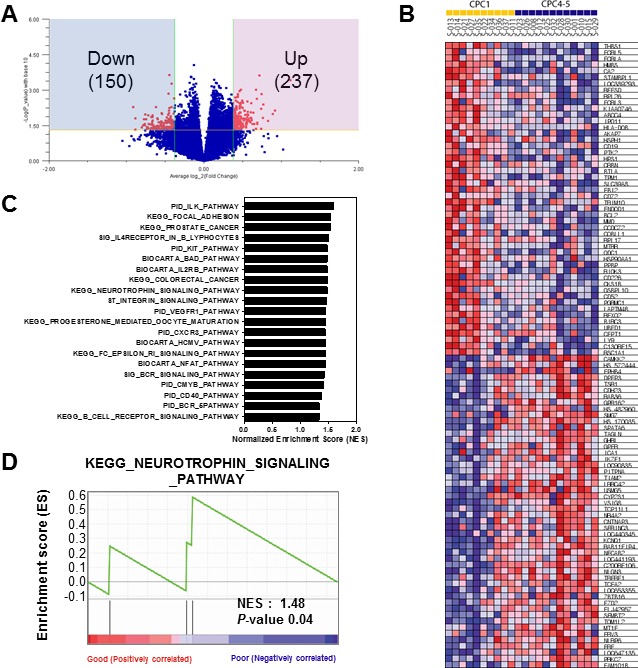
Gene set enrichment analysis of the differentially expressed gene signatures in the CA patients **A**. Volcano plot representation of differentially expressed gene signatures between Good (CPC 1) and Poor (CPC 4-5) cohorts. The number of differentially expressed genes was depicted in the blue and red box. **B**. GSEA corresponding heat map images of the enrichment of the CPC category. Genes in heat maps are shown in rows; a sample is provided in one column. Expression levels are represented as a gradient from high (red) to low (blue). **C**. The bar chart of 21 gene set lists of less than nominal p-value 0.05. **D**. GSEA enrichment plots of KEGG_NEUROTROPHIN_SIGNALING_PATHWAY enriched to neurological outcome is shown; the barcode indicates gene positions. The y-axis indicates the extent of enrichment.

From previous clustering analysis, we detected that CPC 4 and CPC 5 have considerably similar gene expression patterns different with CPC 1 group (Figure 2B and 2C). Therefore, to identify molecular markers that could predict poor CPC score, we combined CPC 4 and 5 into one group, and performed next analyses to select differentially expressed genes for poor CPC score compared with good CPC score group (CPC 1). Briefly, to identify gene expression changes characteristic of poor CPC, we used the Volcano plot method with a stringent cut off value (*p* < 0.05 and 1.3-fold change) to show the poor CPC-related expression data. As shown in Figure 3A, 237 genes were significantly up-regulated and 150 genes were down-regulated in the poor group compared to the good groups. Lists of genes that were down- or up-regulated genes in poor CPC are summarized in Supplementary Table 1. To gain greater insight of the molecular mechanisms associated with the poor CPC molecular markers in patient's blood, we performed gene set enrichment analysis (GSEA) on the differentially expressed genes on genes deregulated by neurological outcome to identify signaling pathways enriched by poor CPC group. Differentially expressed genes were presented as a correlation heatmap and the groups were separated to CPC 1 group (good) and CPC 4, 5 group (poor) (Figure 3B). From GSEA, we charted the 21 gene set lists with nominal *p*-value of less than 0.05 on a bar graph in Figure 3C. In addition, the identification of the specific enriched signaling pathway, which was uncovered because of its characteristic to selectively up-regulate or down-regulate in poor CPC group, could help to understand molecular mechanisms of neurological outcome. As expected, we found the KEGG_NEUROTROPHIN_SIGNALING_PATHWAY gene set was associated with neurological disease. The enrichment plot showed a significantly positive correlation with gene signatures of poor CPC subset in Figure 3D (NES = 1.48, *p* < 0.04).

**Figure 4:**
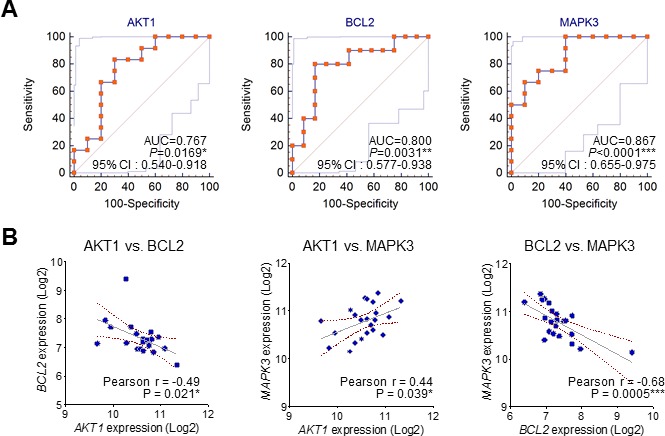
The ROC curve analysis and correlation of the three candidate molecular markers **A**. The receiver operating characteristic (ROC) curves for *MAPK3, BCL2* and AKT1. (AUC: area under the curve; 95% C.I.: 95% confidence interval). **B**. The correlation between the expression of *MAPK3*, *BCL2* and *AKT1* in good and poor outcome groups.

From GSEA, we calculated top 10 gene sets that were ordered by NES rank and draw up gene set lists including each core genes in Table 2. Interestingly, among these core genes of top 10 gene sets, *AKT1*, *BCL2*, and *MAPK3* were commonly enriched in almost gene sets. These findings also suggest that specific molecular markers could respond to gene signatures of neurological outcome in circulating blood of CA patients. Comparative analysis of receiver operating characteristic (ROC) curve was performed to determine whether these candidates were putative biomarkers of poor CPC. The areas under the curve (AUC) of *MAPK3* (AUC, 0.867, *P* < 0.0001), *BCL2* (AUC, 0.800, *P* = 0.0031) and *AKT1* (AUC, 0.767, *P* = 0.0169) were indicated to classify CPC 1 group and CPC 4, 5 group (Figure 4A). The results from the ROC curve analysis indicated preoperative MAPK3 as the strongest independent predictor for neurological outcome. Then, we analyzed the relationship between the expression of *MAPK3*, *BCL2* and *AKT1* in good and poor outcome groups. We notably found that there were significant negative correlations between *AKT1* and *BCL2*, and negative correlations between *BCL2* and *MAPK3*. Furthermore, we found the positive correlation between *AKT1* and *MAPK3* in good and poor neurological outcome group (Figure 4B). Next, to validate the expression of *AKT1*, *BCL2*, and *MAPK3* in the large cohort of cardiac arrest patients, we recapitulated gene expression levels of *AKT1*, *BCL2*, and *MAPK3* in the datasets available from the NCBI, GEO database (GSE29540). Consistently, *MAPK3* gene expression was significantly up-regulated in larger patient cohorts with a poor neurological outcome; however, *BCL2* and *AKT1* were not observed significant expression change. (Supplementary Figure 1)

**Table 2:** Enriched gene set in response to CPC difference by GSEA

No.	**Gene set**			**ES**	**NES**	**NOM p-val**			**GENE**	**Description**
1	PID_ILK_PATHWAY			0.61	1.6	0.01			HSP90AA1	heat shock protein 90kDa alpha (cytosolic), class A member 1
									LIMS1	LIM and senescent cell antigen-like domains 1
									ZYX	zyxin
									AKT1	v-akt murine thymoma viral oncogene homolog 1
2	KEGG_FOCAL_ADHESION			0.48	1.54	0.01			THBS1	thrombospondin 1
									PTK2	PTK2 protein tyrosine kinase 2
									BCL2	B-cell CLL/lymphoma 2
									BIRC3	baculoviral IAP repeat-containing 3
									BIRC2	baculoviral IAP repeat-containing 2
									ZYX	zyxin
									AKT1	v-akt murine thymoma viral oncogene homolog 1
									MAPK3	mitogen-activated protein kinase 3
									ACTN4	actinin, alpha 4
3	KEGG_PROSTATE_CANCER			0.59	1.53	0.02			BCL2	B-cell CLL/lymphoma 2
									HSP90AA1	heat shock protein 90kDa alpha (cytosolic), class A member 1
									AKT1	v-akt murine thymoma viral oncogene homolog 1
									MAPK3	mitogen-activated protein kinase 3
4	SIG_IL4RECEPTOR_IN_B_LYPHOCYTES			0.54	1.5	0.04			BCL2	B-cell CLL/lymphoma 2
									AKT1	v-akt murine thymoma viral oncogene homolog 1
									MAPK3	mitogen-activated protein kinase 3
									STAT6	signal transducer and activator of transcription 6, interleukin-4 induced
5	KEGG_NEUROTROPHIN_SIGNALING_PATHWAY			0.59	1.48	0.04			BCL2	B-cell CLL/lymphoma 2
									AKT1	v-akt murine thymoma viral oncogene homolog 1
									MAPK3	mitogen-activated protein kinase 3
6	KEGG_COLORECTAL_CANCER			0.59	1.48	0.04			BCL2	B-cell CLL/lymphoma 2
									AKT1	v-akt murine thymoma viral oncogene homolog 1
									MAPK3	mitogen-activated protein kinase 3
7	BIOCARTA_IL2RB_PATHWAY			0.59	1.48	0.04			BCL2	B-cell CLL/lymphoma 2
									AKT1	v-akt murine thymoma viral oncogene homolog 1
									MAPK3	mitogen-activated protein kinase 3
8	BIOCARTA_BAD_PATHWAY			0.59	1.48	0.04			BCL2	B-cell CLL/lymphoma 2
									AKT1	v-akt murine thymoma viral oncogene homolog 1
									MAPK3	mitogen-activated protein kinase 3
9	PID_KIT_PATHWAY			0.59	1.48	0.04			BCL2	B-cell CLL/lymphoma 2
									AKT1	v-akt murine thymoma viral oncogene homolog 1
									MAPK3	mitogen-activated protein kinase 3
10	ST_INTEGRIN_SIGNALING_PATHWAY			0.61	1.46	0.04			PTK2	PTK2 protein tyrosine kinase 2
									ZYX	zyxin
									AKT1	v-akt murine thymoma viral oncogene homolog 1

### Clinical predictors of neurological outcomes

A logistic regression analysis model was constructed to rule out the effects of irrelevant factors including several univariate factors. Multiple logistic regression was used to identify clinical covariates that predict a poor neurological outcome. The following covariates were tested: age, sex, witnessed arrest, initial rhythm, time arrest to ROSC, history of previous diseases, cardiac arrest etiology, vital sign (blood pressure, heart rate, respiration rate, temperature) at 48 hr after CA, APACHE II, the level of NSE, *MAPK3, BCL2* and *AKT1* at 48 hr after CA. The level of *MAPK3*, *BCL2*, and *AKT1* were independent predictors of poor neurological outcome (*P* < 0.05). The combined results from the ROC curve analysis and multivariate logistic regression indicated preoperative *MAPK3* as the strongest independent predictor for poor neurological outcome (OR, 1.225, 95% CI, 1.136 to 16.732; *P* = 0.045).

### Biochemical validation of *AKT1, BCL2*, and *MAPK3* expression in the validation cohort

**Figure 5:**
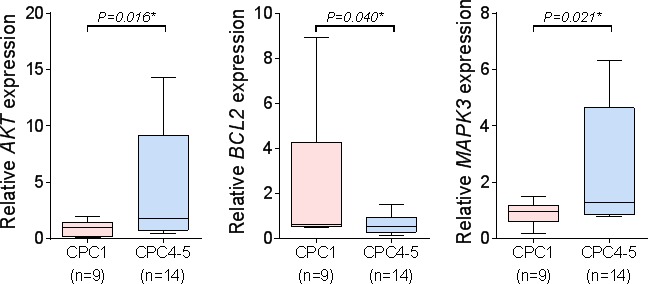
The qRT-PCR of the three candidate molecular markers The qRT-PCR analysis of the three candidate molecular markers, MAPK3, BCL2 and AKT1. (mean ± S.D, *P < 0.05 *versus* CPC1).

In order to validate gene expression data of microarrays and to confirm transcriptional levels of differentially expressed genes, we performed quantitative real-time reverse transcriptase-polymerase chain reaction (qRT-PCR) analysis. As shown in Figure 5, transcriptional levels of three genes (*AKT1, BCL2,* and *MAPK3*) that were up- or down regulated in the validation cohort. Microarray data of *BCL2* gene was down-regulated by 1.46 fold, and *MAPK3* and *AKT1* were up-regulated by 1.31 ∼ 1.35 fold. Results of qRT-PCR were appeared to be under or over-expressed in transcriptional level. Similarly, down-regulated gene (*BCL2*) by 2.97 fold and up-regulated genes (*MAPK3* and *AKT1*) by 3.38∼5.65 fold in qRT-PCR results (Figure 5).

## DISCUSSION

Neurocognitive disturbances are common among survivors of CA. Although initial management of CA, including bystander cardiopulmonary resuscitation and early defibrillation, has been implemented over the last years, few therapeutic interventions are available to attenuate the extent of brain injury occurring after CA. Several studies have been performed to device neuroprotective strategies that target multiple pathways involved in the pathophysiology of postanoxic brain injury [14]. Neurological outcome prediction in comatose patients after CA is important for individual patient care and to help guide end-of-life decision making. It should be recommended that Prognostication focused on end-of-life be delayed beyond 72 hr after CA with accurate use of several prognostic tools.

In several recent studies, RNA extracted from whole blood was used to evaluate RNA expression patterns based on microarrays and showed that total or specific RNA contents were extracted without RNA degradation or inhibited RNA induction in whole blood samples. We used large scale whole genome expression analysis to determine whether gene signatures are associated with comatose patients from CA. We confirmed that transcriptomic analysis of blood cells from patients with good neurological outcome might be distinguished from patients with poor neurological outcome. In the poor outcome group, 237 genes were up-regulated and 150 genes were down-regulated compared with good outcome group. Data-mining approaches through GSEA revealed that differently expressed genes from patients with poor neurological outcome were significantly associated with biological processes linked to *MAPK3*, *BCL2*, and *AKT1*, which are mainly related with the death or apoptosis of neurons and cells.

The occurrence of hypoxic-ischemic encephalopathy after CA was recently integrated in the so-called “post-resuscitation syndrome”, which is characterized by postanoxic brain injury, cardiovascular impairment, and a systemic inflammatory response following the ischemia/reperfusion (I/R) process [18]. Cerebral ischemia is a result of insufficient cerebral blood flow for cerebral metabolic functions. Reperfusion stimulates many pathological mechanisms such as leukocyte infiltration, oxidative stress, inflammation, destruction of blood-brain barrier, platelet activation, nitric oxide release, and apoptosis [19].

At the cell level, the interaction between ligands and receptors regulates a wide spectrum of biological processes *via* the initiation of a complicated cascade of intracellular signaling pathways, like extracellular signal-regulated kinases (ERK), AKT1, mitogen-activated protein kinase (MAPK), PI3γ, protein kinase C, and JAK-STAT [20]. The activation of these signaling pathways leads to cell cycle arrest, cell proliferation, differentiation, tumorigenic development and anti-apoptotic processes [21, 22]. MAPKs are a family of serine-threonine protein kinases, involved in cell growth, differentiation, transformation, and apoptosis. MAPKs play an important role in the transmission of signals from cell surface receptors to the transcriptional machinery in the nucleus. They are activated in response to a variety of extracellular stimuli, including Lipopolysaccharides, hypoxia, and inflammatory cytokine release. The nuclear targets of MAPK signaling pathways are transcription factors, such as activator protein-1 and nuclear factor-kappa B, which regulate the expression of various pro-inflammatory gene expressions. In several studies, ERK 1/2 pathway of the MAPK signal transduction cascade has been heavily implicated in the pathogenesis of post-ischemic neuronal damage and it has been suggested that MAPKs play an important role in the pathogenesis of cerebral ischemia-reperfusion injury. As previously reported, our study using both microarray and qRT-PCR shows that MAPK3 expression is significantly activated in the poor neurological outcome compared with the good outcome. We also observed that MAPK3 was significantly sensitive and specific in ROC analysis providing novel CPC biomarkers (Figure 4).

Cellular Bcl-2 family proteins are known to regulate critical steps in programmed cell death pathway by modulating mitochondrial permeability and function. They are divided on two distinct groups, anti-apoptotic members such as Bcl-2 and Bcl-Xl that prevent cell death and pro-apoptotic members like Bax and Bak [30, 31]. The animal studies of brain ischemia-reperfusion injury showed that the level of Bcl-2 was more decreased in severe brain injury [19, 32]. Like these findings from other studies, our results show that the gene expression of anti-apoptotic protein, Bcl-2 showed greater decreases in poor neurological group compared with good neurological group.

The phosphatidylinositol 3-kinase (PI3K)/Akt1 pathway, which has been extensively studied recently, is a major cell survival pathway that is closely correlated with ischemic brain injury [33]. Akt is a downstream signaling molecule of PI3K, which serves key roles in mediating anti-apoptotic actions [34]. Three genes encoding Akt (akt1, akt2, and akt3) have been identified in mammalian genomes. Akt1 is widely expressed in the brain and its activation is correlated with phosphorylation of Ser473 at the C terminus. Akt1 phosphorylation at Ser473 is closely related to neuronal survival and plays an important role in neuronal protection in ischemic disease of the central nervous system [35, 36]. Studies of a cerebral ischemia-reperfusion (I/R) animal model demonstrated that neuroprotective effects were inhibiting apoptosis *via* activation of the PI3K/Akt1 signaling pathway. However, our results were not in accordance with the reported results; the poor neurological outcome group showed a greater increase in the level of Akt1 compared with good outcome group. The reason of these different results may be may be due to different study designs. Other studies were performed in the hippocampus part of the brain, while our study examined circulating blood. In a neuroinflammation mouse model generated using LPS, the phosphorylation of the level of Akt1 was measured in different parts of the brain. They demonstrated different changes on Akt1 signaling in major mouse brain regions in response to LPS. The Akt1 level was significantly increased in the striatum, no changes in the cortex and hypothalamus, and an increase in hippocampus although no significant difference was found compared with the control mice [39]. Since most of cerebral I/R animal model studies were performed in the brain tissue, especially in the hippocampus, further studies looking at the level of Akt in blood are needed.

Since early prognostication after CA is difficult, several prognostic tools with clinical examination are recommended to improve the accuracy of prognosis. TTH makes early prognosis more complex, mainly due to the liberal use of sedatives, analgesics, and muscle relaxants during TTH. The most of clinically used prognostic tools are subject to inter-observer variability. Therefore, research into biomarkers that require less expertise, less inter-observer variability, and are more readily repeated has been performed. NSE is a biomarker clinically used for prediction of outcomes after CA, but its use has been limited by a lack of standardization, as well as diverging cutoff levels for prediction of poor prognosis in patients [9–13]. Identification of new biomarkers and use of other prognostic tools may improve the accuracy of prognosis of neurological recovery.

Our study has some limitations. First, this study was performed in a single tertiary referral center with a small sample size. The number of patients enrolled in this study was small, resulting in wide confidence intervals for sensitivity and specificity. Second, the long period between samples collection and analysis of the samples might have affected the quality of the samples. Fortunately, mRNA is known to be stable both at room temperature and after repetitive cycles of freeze-thawing and was stored at −80°C until analysis, and samples were thawed only once. Third, we obtained mRNA data at one time point only. Initially, we designed this as a preliminary study, and chose the time point that appeared best to accurately evaluate biomarkers in cardiac arrest. Further studies using larger populations of cardiac arrest patients in multiple centers are needed to determine the kinetics of mRNA levels after CA both hypothermia and normothermia.

In this preliminary study, we evaluated whether we could reliably detect differences in gene expression between good and poor neurological outcome, and whether we could detect biomarkers that predict neurological outcome. We verified that the gene expressions of circulating blood cells of comatose patients after CA were significantly different between good and poor neurological outcome group. These different biosignatures allowed the identification of *MAPK3*, *BCL2*, and *AKT1 KT*as predictors of neurological outcome in comatose patients after CA.

Finally, this study suggests that characteristic molecular signatures in the circulating blood of comatose patients after CA could be useful surrogate markers to predict neurological outcome. Further studies on these gene signatures are needed to understand the mechanisms triggering the development of CA and effects of each of these molecules following CA.

## MATERIALS AND METHODS

### Patients and sample collection

Comatose patients successfully resuscitated from CA at a single tertiary center between March 2013 and Feb 2014 were enrolled into this study. Computed tomography of the brain was performed liberally at admission to exclude patients with intracranial bleeding. Patients who died within 72 hours after CA, were under 18 years, experienced traumatic cardiac arrest, severe irreversible brain damage, had a known severe neurological diseases and terminal malignancy were excluded. Informed consent was provided according to the Declaration of Helsinki. Written informed consent was obtained from all subjects. Histological assessment was previously described. The study was approved by the Institutional Review Board of Seoul St. Mary's Hospital (XC12TIMI0075D).

All patients after ROSC were treated according to current recommendations [6]. Before the induction of TTH in comatose patients, sedation with midazolam (0.08 mg/kg intravenously) and paralysis with rocuronium (0.8 mg/kg intravenously) were administered for shivering control, followed by continuous infusion of midazolam (0.04-0.2 mg/kg/h) and rocuronium (0.3-0.6 mg/kg/h). A target temperature of 33°C was maintained for 24 hr. After the completion of the TTH, rewarming was performed at a rate of 0.25°C/h until the patient's temperature reached 36.5°C. Sedation and paralysis were reduced during rewarming and were discontinued until the central temperature reached 35°C.

Blood samples for total RNA extraction were collected from patients at 48 hr after CA and collected in blood RNA tubes (PAXgene, Qiagen BD Company, UK) for RNA isolation. Total RNA extracted from PAXgene™ tubes was used to study gene expression by microarrays.

### Neurological outcomes

All patients were categorized at discharge according to the five-point Cerebral Performance Category (CPC) scale: CPC score 1: good cerebral performance, CPC score 2: moderate cerebral disability, CPC score 3: severe cerebral disability, CPC score 4: coma and CPC score 5: death [40]. A CPC score of 1 or 2 after CA was considered a good neurological outcome, CPC score of 3, 4, or 5 is a poor neurological outcome. Both at discharge and at six months after resuscitation, neurological outcome was evaluated by the authors *via* a telephone interview.

### RNA isolation and blood whole genome expression analysis

Total RNA was extracted from blood samples according to optimized methods previously described, with slight modifications. Briefly, whole blood (2.5 mL per patient) was collected directly into PAXgene Blood RNA tubes, labeled with a unique identification number, stored at room temperature, and transferred to the laboratory within four hours for blood processing. Total RNA was extracted from blood samples using the PAXgene blood RNA kit (Qiagen) and purified with RNeasy kit (Qiagen) according to the manufacturer's instruction. The quality of total RNA was analyzed with the RNA StdSens Chips on the Experion™ system (BioRad, Hercules, CA). Microarray analysis was performed using Sentrix HumanRef-6 Expression BeadChip or HumanHT-12 v3 Expression BeadChip (Illumina, Inc., San Diego, CA). Approximately 37,000 genes with unique probe IDs were common to both platforms and were used for combined data analysis. The RNA was processed with Illumina RNA Amplification Kit (Ambion, Inc., Austin, TX) according to the manufacturer's instructions starting with 800 ng total RNA. Resulting biotin-labeled cRNA was recovered and purified with RNeasy kit (Qiagen), hybridized to the beadchips, and fluorescently tagged and scanned with Illumina BeadStation (Illumina) according to the manufacturer's protocol. All arrays were performed in the same core facility.

### Blood transcriptome data analysis

Data analysis for blood transcriptome from CA patients was performed using the following softwares: GenomeStudio (version 3.0, Illumina), GenPlex™ (version 3.0, ISTECH, Inc., Seoul, Korea), EXCEL (Microsoft), and GSEA (version 2.07, Broad Institute). Briefly, GenomeStudio (version 3.0) was used for the data acquisition and calculation of signal values on Illumina expression beadchip. Normalization of expression data and hierarchical clustering was performed by GenPlex™ (version 3.0). For primary data filtering, spots with a P-call (Detection call *P*-value < 0.1) were selected, and normalized *via* quantile normalization. A multitude of analyses was performed using the normalized and filtered data. Sets of differentially expressed genes (DEGs) were identified by combination analysis of Welch's *t* test and fold change, and the DEGs with a fold change deregulation of more than 1.3 and *P*-value < 0.05 were selected.

### GEO data analysis

To analyze the expression level of AKT1, BCL2, and MAPK3, mRNA expression data sets were obtained from the National Center for Biotechnology Information (NCBI) Gene Expression Omnibus (GEO) database (Accession No. GSE29540)

### Molecular pathway mining and gene set enrichment analysis

To investigate CA-specific genes that are enriched into the known molecular databases, Gene Set Enrichment Analysis (GSEA) was conducted using the standard procedures (http://www.broadinstitute.org/gsea). Briefly, the set of microarray data was analyzed for enrichment in Canonical pathways gene sets including 1,330 gene sets related to the cellular component and biological pathway. The GSEA method was used with the dataset collapsed to gene symbols, 1,000 permutations and phenotype permutation type, and Pearson metric for ranking genes. As the output, GSEA provides a nominal *P* value for each gene set, which represents how significantly up- or down-regulated the genes within that set are in the microarray data comparison.

### RNA isolation and quantitative real-time polymerase chain reaction (qRT-PCR)

Using RNA isolated from blood as a template, a tetro cDNA synthesis kit (Bioline USA Inc., Tounton, MA, USA) was used to synthesize cDNA. For qRT-PCR analysis, reactions were conducted with SensiFAST^TM^ SYBR^®^ No-ROX kit (Bioline USA Inc., Tounton, MA, USA). The level of GAPDH was used as a loading control. The real time PCR was monitored using the CFX96^TM^ Real-Time System (BioRad, Hercules, CA) that allowed checking of the threshold cycle (Ct): the exponential amplification time of PCR products. Results are displayed as the mean values from triplicate experiments. Relative expression values were normalized to control -2^-(Target Ct-Control Ct)^. The primer sequences for MAPK3 were purchased from Bioneer (Daejeon, Korea), BCL2 primer sequences were 5′-GCTGGACGATAGCTTGGA-3′ (forward) and 5′-GATGACAGATAGCTGGTG-3′ (reverse) and AKT1 primer sequences were 5′-CCTGTGGATGACTGAGTACCTGAA-3′ (forward) and 5′-GGGCCGTACAGTTCCACAAA-3′ (reverse).

### Statistical analysis

All statistical analyses were performed with the MedCalc version 12.1.4.0 (MedCalc Software, Mariakerke, Belgium). Receiver operating characteristic curve (ROC) analysis was constructed to assess the sensitivity and specificity of blood biomarkers and to determine the ability of the various parameters to discriminate CA patients with poor CPC score. The method of DeLong et al., was used for the calculation of standard error of the areas under the ROC curves (area under the curve, AUC) and of the difference between two AUCs [41]. *P*-values less than 0.05 were considered statistically significant. Continuous variables are reported as mean and interquartile range. Categorical variables are reported as number and percentages. Univariate and multivariate logistic regression analyses were performed to identify independent predictors for good or poor prognosis patients. All predictor variables that were identified as significant at a two-tailed nominal probability value of less than 0.05 in univariate regression analyses were entered into a multivariate logistic regression analysis model.

### Data access

Expression profiling data has been deposited in the Gene Expression Omnibus under accession code: GSE92696.

## SUPPLEMENTARY FIGURES


