# Supplementary material for: Identification of novel biomarkers for prediction of neurological prognosis following cardiac arrest
Source: Oncotarget. 2017 Jan 28;8(10):16144–57. doi: 10.18632/oncotarget.14877 (PMC5369953; doi:10.18632/oncotarget.14877)
Supplement: Supplementary file 1 [file oncotarget-08-16144-s001.pdf]

Identification of novel biomarkers for prediction of neurological prognosis following cardiac arrest

Supplementary Figure 1

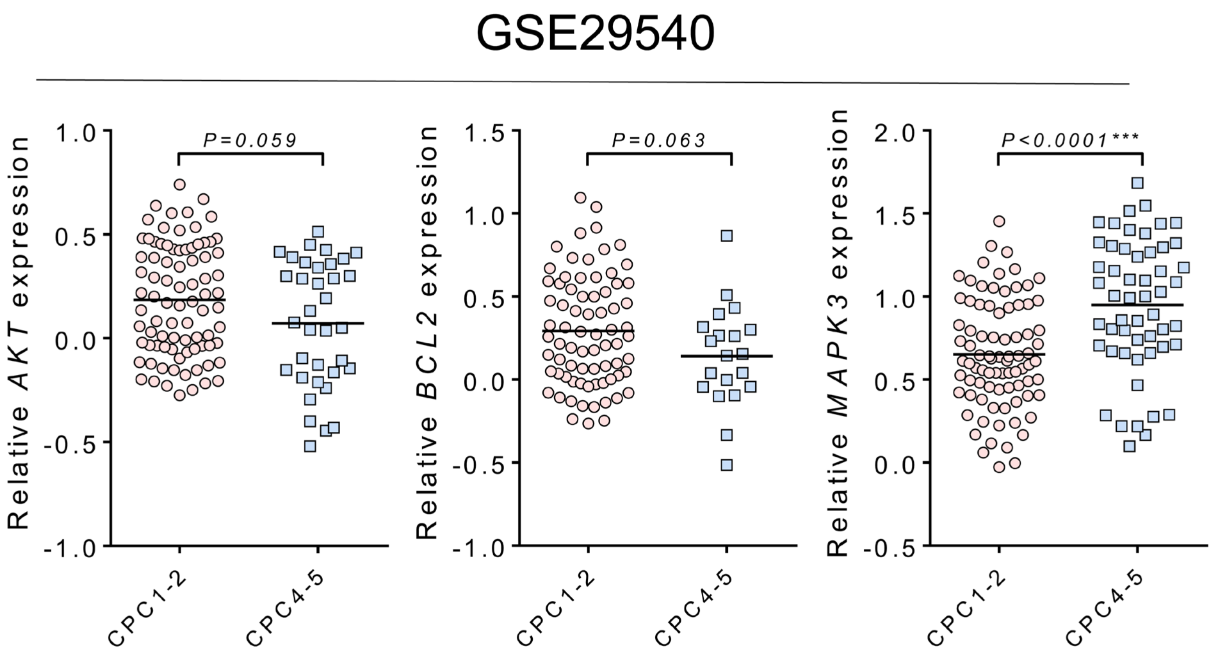

To validate the expression of *AKT1*, *BCL2*, and *MAPK3* in the large cohort of cardiac arrest patients, we recapitulated gene expression levels of *AKT1*, *BCL2*, and *MAPK3* in the datasets available from the NCBI, GEO database (GSE29540)
